# Delivering What We PROMISED: Outcomes of a Coaching and Leadership Fellowship for Mentors of Underrepresented Mentees

**DOI:** 10.3390/ijerph18094793

**Published:** 2021-04-30

**Authors:** Marie K. Norman, Colleen A. Mayowski, Steven K. Wendell, Michael J. Forlenza, Chelsea N. Proulx, Doris M. Rubio

**Affiliations:** 1 Department of Medicine, Institute for Clinical Research Education, University of Pittsburgh School of Medicine, Pittsburgh, PA 15213, USA; mkn17@pitt.edu (M.K.N.); cnp10@pitt.edu (C.N.P.); dmr18@pitt.edu (D.M.R.); 2 Department of Pharmacology and Chemical Biology, University of Pittsburgh School of Medicine, Pittsburgh, PA 15213, USA; wend0017@pitt.edu (S.K.W.); mjf@pitt.edu (M.J.F.); 3 Coaching through Stressful Experiences, Pittsburgh, PA 15213, USA

**Keywords:** diversity, equity, inclusion, mentoring, career coaching

## Abstract

Research demonstrates that mentorship can significantly improve career success, career satisfaction, and persistence for underrepresented (UR) minority faculty. However, many UR faculty members do not receive the mentorship they need, nor do mentors always possess the range of skills required to guide UR mentees through the unique challenges they face. We developed a 1-year fellowship training program, PROMISED, designed to help mentors promote career self-authorship and leadership among their UR mentees. PROMISED fellows participated in a two-day in-person training to develop career coaching skills, followed by a series of one-month leadership training/mentoring modules. We assessed mentors’ skills at the start and completion of the program. We found that PROMISED fellows reported an increase in perceived skill level in nearly every training topic, with “addressing diversity” demonstrating the most significant change. These results provide evidence that career coaching and leadership training offer an effective supplement to traditional mentor training and that mentors can incorporate these skills effectively into their mentoring practice. Taken together, we believe our data suggest that a program designed to train mentors in coaching and leadership can enhance career satisfaction, persistence, and retention of their UR mentees.

## 1. Introduction

A diverse workforce improves the quality of biomedical research and clinical care [1,2], yet despite calls over several decades to diversify the workforce in academic medicine [3,4,5], progress has been incremental. Representation of underrepresented (UR) faculty in academic institutions, in particular Black individuals or African Americans, Hispanics or Latinos, American Indians or Alaska Natives, Native Hawaiians and other Pacific Islanders, remains low [6], particularly at the senior faculty level [7], and attrition [8,9,10] continues to be unacceptably high [6].

Mentorship has been demonstrated to significantly improve career success, career satisfaction, and persistence for UR researchers [11,12,13,14]; however, many do not receive the mentorship they need [10,13,14,15,16,17]. Indeed, even where mentors are available, they may not possess the necessary skills to be effective. Research suggests the need for robust mentor training programs, in particular for mentors working with diverse mentees [18] that recognize the need for multiple forms of mentoring to guide UR researchers through the unique challenges they face [15].

Career coaching may be an especially effective supplement to traditional mentoring and is increasingly being utilized along with mentoring to support professional development for trainees. Career coaching represents a substantively distinct and complementary approach to academic mentoring. Its methods and supporting skills are primarily experiential and behaviorally experimental and are consistent with a developmental, action-learning approach. Compared to mentoring, career coaching is relatively more facilitative than directive and seeks to develop the trainee’s agency, self-efficacy, and perspective-taking while honoring their unique qualities, personality, and life experiences. Combined with the development of academic and professional competencies (the primary focus of mentoring), the development of these capacities in UR trainees increases the likelihood of their retention and success.

We contend that supplementing more traditional mentor training with training in professional coaching skills such as mindfulness, being fully present, deep listening, and powerful questions enhances the quality and experience of mentoring for both mentors and trainees. It shifts the focus from one of developing only technical expertise to one that is additionally more interpersonal and mentee-centric.

Increasing this mentee-centric focus is even more important considering the evolving 21st century career landscape of increased mobility and career switching. To take advantage of new and diverse career pathways that are emerging, mentors will need to support the development of career adaptability in UR mentees. The reflective career exploration tools and career planning strategies employed in professional career coaching offer valuable enhancements to prior mentor training in support of UR career adaptability. We therefore sought to complement prior mentor training with training in the basic skills of professional career coaching.

In addition to supplemental training in career coaching, mentors need explicit training in leadership skills. Teaching mentors leadership skills can provide them with a more holistic, 360-degree perspective of the knowledge needed to develop a productive career. In particular, leadership skill training can help mentors guide mentees through elements of the “hidden curriculum”: skills and knowledge critical for successfully establishing and maintaining an academic career that are not always taught as part of the formal curriculum. For example, if mentors understand the “academic biomedical research code,” then they can better guide their mentees’ path along the career pipeline. If mentors learn to develop a strategic plan for their own careers, they can better support their mentees to develop one for theirs.

In an effort to retain UR junior investigators and promote diversity in the workforce, we developed a novel mentor training program called PROMISED (PROfessional MentorIng Skills Enhancing Diversity), which was funded as a supplement from the National Research Mentoring Network (NRMN). The PROMISED program sought to identify mentors devoted to mentoring UR junior investigators and teach them basic career coaching and leadership skills. We hypothesized that mentors who learned and implemented these skills would more effectively facilitate the career development of UR mentees.

## 2. Materials and Methods

We developed a year-long fellowship for mentors committed to mentoring people from underrepresented backgrounds. The fellowship was composed of a two-day in-person training focused on developing career coaching skills. Following the workshop, participants selected and engaged in six (out of 13) online month-long modules designed to improve their leadership skills. Topics for these modules were drawn from popular leadership training programs that had been offered at the University of Pittsburgh (see Table 1). Fellows were recruited by advertising broadly across the country, primarily through the NRMN, Clinical and Translational Science Consortium, and the University of Pittsburgh. The applications were reviewed by the PROMISED leadership team and participants were selected based on their commitment regarding mentoring someone from an underrepresented background. Although fellowship applicants were required to demonstrate an interest in mentoring trainees from UR backgrounds, they were not required to be UR as race has been shown to have little discernable effect on mentoring relationships at the professional level [18,19]. Nearly all applicants were accepted except those who were (1) not faculty; (2) not in a position to mentor; or (3) not able to make the time commitment. We had two consecutive cohorts participate in the fellowship (2016–2017 and 2017–2018).

### 2.1. Career Coaching

The PROMISED career coach training included 14 training hours over two days. This training was developed and facilitated by two International Coach Federation (ICF) certified professional coaches with PhD backgrounds in biomedical and behavioral research. The content covered two primary domains: career management and professional coaching skills. The career management domain focused on changes in the 21st century career landscape, career adaptability, career decision making, reflective career assessment and exercises, and career planning tools. The professional coaching skills domain included mindfulness and awareness monitoring, deep listening, presence and curiosity, and powerful questions for reflective thinking.

Participants probed their own career goals by completing the reflective career assessments (e.g., career preferences, personal interests, and skill assessment) followed by group discussion and debriefing. The professional coaching skills were practiced in multiple rounds of exercises in triads.

### 2.2. Online Modules

After participating in the career coaching workshop, participants were provided with the list of 13 online modules and were invited to select six (see Table 1). This allowed us to keep class sizes small and foster a more intimate environment and richer interactions. Each module lasted one month and was composed of both synchronous and asynchronous components. Asynchronously, participants watched short videos, read relevant materials, and engaged with one another in online discussion forums. Instructors then held weekly synchronous meetings for 1.5 h in which participants took part in lively discussion about the materials for that week’s topic.

The majority of the instructors were faculty at the University of Pittsburgh. We had an additional instructor from the University of Colorado, as she was faculty previously at Pittsburgh, but was recruited to Colorado at the start of the grant. The instructors were a blend of PhDs and MDs. They represented various disciplines (medicine, social work, infectious disease) and were at different career stages, spanning assistant professor through professor. Two were African American, two were white, and one was Asian American. Four of the five instructors were women.

In order to develop the modules, the PROMISED instructors worked as a team, led by an online education expert. She developed templates which instructors used to design their module content. Instructors found that much of the content, including videos, could be curated from the internet, allowing us to repurpose existing material for our modules, and eliminating the need to create entirely new material for every module. After each module was offered, the instructor would receive feedback from the fellows. The instructor would then work with the online education expert to review the feedback and make adjustments to the module. Most of the adjustments were minor: suggestions for replacing videos, revising discussion board prompts, or decreasing the amount of homework.

### 2.3. Assessment

*Career Coaching***.** As part of the assessment of the career coaching training, PROMISED fellows completed a modified version of the Mentoring Competency Assessment (MCA) [20]. These modifications included removal of several items and rewording of other items for greater consistency with the language and philosophy of a professional coaching approach. These modifications were made by an ICF certified coach and instructor to align with the facilitative end of the mentoring continuum and the ICF core competencies. For example, changing wording from “Setting Career Goals” to “Working with mentees to set career goals” and “Setting clear relationship expectations” to “Working with mentee to set clear expectation of the mentoring relationship” are more mentee-centric orientations that supports their autonomy and self-efficacy. Removal of questions such as, “Estimating mentee ability” and “Helping network effectively” reduces the directive and analytical orientation.

Participants completed the modified MCA prior to training and immediately following the training. All pre- and post-data were matched: if an individual was missing an item response for an item on the pre-test, their response on the post-test was excluded as well and vice versa.

Participants were also invited to complete a career coaching training feedback survey immediately following the conclusion of the workshop to rate various aspects of the training and to respond to open-ended questions. Lastly, the mentors were invited to complete a Mentoring Exit Survey following the conclusion of their year-long fellowship. The survey included several questions to reflect on their perspectives about the career coach training and the extent they have utilized the career coaching skills in mentoring.

*Online Modules*. At the completion of each module, participants received a module evaluation in which they rated the instructor and the course in terms of effectiveness, relevant course content, workload, and whether they would recommend the module to another person. Participants were also asked to provide comments as to (1) what aspects of the module they can apply to their career and (2) any suggestions to improve the module.

At the end of the fellowship, participants were asked to rate which modules were most beneficial for them. They were also asked to assess their level of skill with regard to leadership, professional development, multidisciplinary teamwork, communication, and management techniques.

### 2.4. Evaluation and Data Analysis

We used formative and summative evaluation for the program evaluation. Formative evaluation was used to provide feedback to the certified career coaches and instructors so that they could revise and improve the training as needed. We present the summative evaluation in this manuscript to demonstrate the impact of the fellowship on the PROMISED fellows.

Descriptive statistics were used to show the distribution of responses across all variables for each cohort as well as combined. To test the change in career coaching and leadership skills, we used paired t-tests. All analyses were conducted using Stata version 14.2.

## 3. Results

### 3.1. Participants

Across both cohorts, we had a total of 61 participants who completed the assessments, of which 74% were female. Fifty-one percent were from an underrepresented background (i.e., Hispanic, Hawaiian, or Black/African American). The majority of the participants were at academic institutions across various ranks (18% Assistant Professor; 57% Associate Professor; and 15% Professor). Several participants held leadership roles at their institutions such as dean or associate dean, center director, program director, or division chief. The geographic distribution of the participants was quite diverse across the continental United States, as well as Hawaii and Puerto Rico.

### 3.2. Career Coaching

For the modified MCA, participants in the training were asked in both the pre-training and post-training to “Please rate how skilled you feel you are in each of the following areas”. Ratings utilized a seven-point Likert-type scale with 1 = “not at all skilled”, 4 = “moderately skilled”, 7 = “extremely skilled”, and the option for “N/A” when not applicable. Individuals who answered with an “8” or “N/A” on either the pre or post-test also had the other response on the item excluded (if both pre and post-test were not “8” or “N/A”).

In looking at the differences on the modified MCA, we found an increase in all of the items except *Constructive Feedback*, which essentially did not change (see Table 2). We summed the items for a score total to test for overall improvement and found a significant improvement from pre to post (t = 3.63; *p* ≤ 0.001). To minimize Type I Error, we then tested the changes in the five items with the largest differences and all of them had a significant improvement. *Taking into account the biases and prejudices you bring to the mentor/mentee relationship* had the largest differences, increasing from 4.07 to 4.97 (t = 4.36; *p* ≤ 0.001). This is interesting to note as we did not do implicit bias training; rather, the career coaching was focused on meeting your mentee where they are and not making any assumptions about them. The second highest improvement was *Working with mentee to set clear expectation of the mentoring relationship* (this changed from 4.08 to 4.78; t = 4.07; *p* < 0.001). This item was not surprising given that expectation-setting was a major focus of the career coaching. The third largest improvement was *Working effectively with mentees whose personal background is different from your own*, which went from 4.50 to 5.17 (t = 4.02; *p* < 0.001). As noted above, the career coaching focused on honoring individual differences.

A few items did not change appreciably, as noted in the table. Of note is *Active Listening* which was explicitly taught in the Career Coaching. While a difference is noted in the median, the mean did not increase dramatically.

### 3.3. Online Modules

In general, participants valued the modules. Across all of the modules, 88% either Strongly Agreed or Agreed that the module content was appropriate to their needs (range 73–100%) and 90% either agreed or strongly agreed that they would recommend the module to a colleague (range 68–100%). Participants rated *Difficult Conversations* and *Creating a Five-Year Plan* as being the most beneficial of the modules. Some of the comments from these modules include “This was a very useful module that I see myself using in all aspects of my life”. “This was an outstanding module and especially helpful to me. I am already using the things I learned”. “Upon reflection, I can see where having a 5-year career plan is of critical importance”. “It helps me strategize and be more intentional with my career path”.

*Understanding Academia* and *Understanding the Code* were among the lowest ranked modules; however, even in these two modules, 89% and 91% Strongly Agreed or Agreed that the module content was appropriate to their needs, respectively. Moreover, 89% and 91% Strongly Agreed or Agreed that they would recommend the module to a colleague. Some comments associated with these modules include “This module was excellent for pushing me to contemplate and better appreciate some of the important administrative and leadership decisions occurring at my university”. “Understanding what occurs behind the scene was critically important to me. I’m very pleased with the transformative information that I’ve received”. *Leading Your Research* was ranked the lowest. However, the scores for “appropriate for my needs” and “would recommend to a colleague” improved dramatically between the first and second cohort (60% vs. 92%; 40% vs. 92%, respectively), indicating that the improvements to the module were well received.

## 4. Conclusions

We theorized that if mentors developed career coaching and leadership skills, they would be more effective mentors and therefore better facilitate the careers of UR mentees. To test this theory, we developed a year-long fellowship for mentors who expressed a commitment to mentoring faculty from underrepresented backgrounds. We found that training mentors in career coaching provided them with a valuable supplemental skill set, useful for helping mentees gain a sense of ownership over their careers. Mentors felt able to incorporate these skills into their mentoring to enhance the mentoring experience for their mentees. Our online leadership modules also led to a measurable increase in self-reported leadership skills across all domains.

It is important to note that professional career coaching is fundamentally different from traditional mentoring. Rather than diagnosing issues and advising solutions, the coach acts as a facilitator to elicit the mentee’s exploration and self-discovery. Ultimately, this empowers mentees to self-author their own authentic career vision, which is critical for career satisfaction and also fosters persistence in their research careers. The impact of coaching on academic and career planning has been studied in undergraduate students across a range of public, private, and proprietary institutions and has been found to positively impact retention and completion rates. In fact, the impact of coaching is more sustained than financial incentives [21]. Our results indicate that our career coaching training did improve the skills of the mentors. While we did not follow the mentees over time, as this was not part of the original project, we speculate that this training will positively impact mentees by enabling them to drive their own careers, leading them to develop a career that meets their personal definition of success. Unfortunately, we have been unable to continue to offer the fellowship due to the expiration of the grant; however, we have disseminated the training in multiple ways, so the effect of the program is ongoing. When training other groups of mentors, the results are similar to those evaluated during PROMISED: the mentors express that the career coaching elements brought considerable value to their mentoring. In another collaboration with nine Minority Serving Institutions, participants found the training so valuable they asked for *additional* instruction in career coaching. One institution asked us for the entire online module curriculum, which they are now offering at their own institution.

These results indicate success; however, our findings are not without limitations. For example, it is important to note that the exit survey was completed with only the first cohort, and our sample size limited us in breaking out subgroups for analysis. Ideally, the PROMISED fellows would need to be assessed longitudinally to examine mentor behavior change over time. These questions warrant further analysis. Moreover, these results do not provide direct evidence of improvements to workforce diversity. However, we believe they suggest that career coaching and leadership training can be an effective supplement for mentors working to develop mentees’ ownership over their careers, thus contributing to persistence in the field. These results also show that mentors can incorporate these skills into their mentoring practice. We also found evidence that providing mentors with leadership training can enhance mentoring such that the mentor can more ably help mentees with leadership development. Taken together, we believe our data show that a program designed to train mentors in coaching and leadership can enhance career satisfaction/persistence/retention of their UR mentees.

The existence of the leaky career pipeline for biomedical researchers is well documented [8,9,21,22,23,24], as is the lack of racial and ethnic diversity in the biomedical workforce [8,22,24]. Research demonstrates that those from underrepresented backgrounds encounter even more deterrents than those from majority backgrounds as they attempt to progress through their career [24]. We know, too, that mentors play an important role in retaining UR researchers [21,23,24]. However, the pervasive problem of underrepresentation suggests that UR investigators may require supplemental career development and support beyond what many mentors are able to offer [24]. Providing career coaching training to mentors of UR mentees could be an effective approach to helping mentees persist in their careers.

## Figures and Tables

**Table 1:** Modules for leadership skill development.

Topic	Leadership Competency	Description
Understanding Academia	Leadership, Ethics and Professional Norms	learn about organizational structures, leadership styles, role expectations, and budgets
Strategic Planning	Leadership	define mission, vision and goals and learning how to apply strategic planning to a achieve goals
Setting the Culture	Professional Norms	expectations and guidelines that contribute to the success or failure of that group
Leading Your Research	Leadership	how to get recognition for your research, make your team more visible, and help your mentees follow this path
Understanding Strengths and Weaknesses	Leadership, Multidisciplinary Teamwork	creatively working with weaknesses, leveraging strengths, and the powerful solutions
Managing Others	Management	focus on an individual’s management style, job descriptions, helping others prioritize, and evaluation and feedback
Understanding the Code	Leadership	discuss three types of codes—environmental, historical, and cultural—and ways to effectively operate within them
Creating a Five-Year Plan	Leadership, Written Communication	break down the steps to career planning by identifying goals and the steps needed to meet those goals
Executive Shadowing	Leadership, Management, Oral Communication	(1) Pre-shadowing training where you will learn your elevator speech; (2) Shadowing where you will shadow someone in leadership; and (3) Post-shadowing debriefing where we will reflect on the experience and summarize take-away key points
Difficult Conversations	Oral Communication, Management	develop strategies to successfully navigate through difficult conversations and be able to help mentees do the same
Leading Diverse Teams	Leadership, Multidisciplinary Teamwork	how to increase self-awareness of diversity and how to manage diverse teams
Time Management	Management	build new time management strategies and learn about tips and tools to become more efficient, effective and satisfied

**Table 2:** Summary statistics for baseline and post-workshop data.

	Combined Summary Statistics						
	Pre-Test			Post-Test			Difference in Means
Question	Med	Mean	SD	Med	Mean	SD	
Active Listening	5.00	5.12	1.14	5.50	5.25	1.17	0.13
Constructive Feedback	5.00	5.00	1.08	5.00	4.95	1.01	−0.05
Establishing an effective relationship	5.00	5.05	1.23	5.00	5.26	1.11	0.21
Identifying and accommodating different communication styles	4.00	4.31	1.12	4.00	4.56	1.34	0.25
Employing strategies to improve communication with mentees	4.00	4.28	1.05	5.00	4.79	1.24	0.51
Coordinating effectively with mentee’s other mentors	4.00	4.17	1.25	4.00	4.43	1.68	0.26
Working with mentee to set clear expectation of the mentoring relationship	4.00	4.08	1.24	5.00	4.78	1.39	0.70 *
Aligning your expectations with your mentees’	4.00	4.26	1.33	5.00	4.79	1.27	0.53
Considering how personal and professional difference may impact expectation	5.00	4.60	1.33	5.00	5.18	1.31	0.58 *
Working with mentees to set career goals	5.00	5.10	1.23	5.00	5.28	1.23	0.18
Helping mentees develop strategies to meet career goals	5.00	4.69	1.34	5.00	5.03	1.08	0.34
Motivating your mentees	5.00	4.67	1.31	5.00	5.18	1.26	0.51
Building mentees’ confidence	5.00	5.08	1.24	5.00	5.32	1.16	0.24
Stimulating your mentees’ creativity	5.00	4.45	1.26	5.00	5.03	1.20	0.58 *
Negotiating a path to professional independence with your mentee	4.00	4.26	1.33	5.00	4.82	1.15	0.56
Taking into account the biases and prejudices you bring to the mentor/mentee relationship	4.00	4.07	1.35	5.00	4.97	1.34	0.90 *
Working effectively with mentees whose personal background is different from your own	5.00	4.50	1.40	5.00	5.17	1.29	0.67 *
Helping your mentees balance work with their personal life	5.00	4.39	1.53	5.00	4.81	1.31	0.42
Understanding your impact as a role model	5.00	4.69	1.47	5.00	5.24	1.33	0.55
Helping your mentees’ acquire resources	4.50	4.38	1.40	5.00	4.77	1.43	0.39

* Top 5 with most improvements *p* < 0.01.

## Data Availability

The data presented in this study are available on request from the corresponding author. The data are not publicly available due to privacy protections for the study participants.
